# High-resolution projections of outdoor thermal stress in the twenty-first century: a Tasmanian case study

**DOI:** 10.1007/s00484-024-02622-8

**Published:** 2024-03-01

**Authors:** Ben Weeding, Peter Love, Kathleen Beyer, Arko Lucieer, Tom Remenyi

**Affiliations:** 1 School of Geography, Planning, and Spatial Sciences, University of Tasmania, Sandy Bay, TAS 7001 Australia; 2 Climate Futures Research Group, University of Tasmania, Sandy Bay, TAS 7001 Australia; 3 Acclimatised Pty Ltd, Blackmans Bay, TAS 7052 Australia

**Keywords:** Bioclimatology, Thermal stress, Multivariate bias correction

## Abstract

To adapt to Earth’s rapidly changing climate, detailed modelling of thermal stress is needed. Dangerous stress levels are becoming more frequent, longer, and more severe. While traditional measurements of thermal stress have focused on air temperature and humidity, modern measures including radiation and wind speed are becoming widespread. However, projecting such indices has presented a challenging problem, due to the need for appropriate bias correction of multiple variables that vary on hourly timescales. In this paper, we aim to provide a detailed understanding of changing thermal stress patterns incorporating modern measurements, bias correction techniques, and hourly projections to assess the impact of climate change on thermal stress at human scales. To achieve these aims, we conduct a case study of projected thermal stress in central Hobart, Australia for 2040–2059, compared to the historical period 1990–2005. We present the first hourly metre-scale projections of thermal stress driven by multivariate bias-corrected data. We bias correct four variables from six dynamically downscaled General Circulation Models. These outputs drive the Solar and LongWave Environmental Irradiance Geometry model at metre scale, calculating mean radiant temperature and the Universal Thermal Climate Index. We demonstrate that multivariate bias correction can correct means on multiple time scales while accurately preserving mean seasonal trends. Changes in mean air temperature and UTCI by hour of the day and month of the year reveal diurnal and annual patterns in both temporal trends and model agreement. We present plots of future median stress values in the context of historical percentiles, revealing trends and patterns not evident in mean data. Our modelling illustrates a future Hobart that experiences higher and more consistent numbers of hours of heat stress arriving earlier in the year and extending further throughout the day.

**Supplementary Information:**

The online version contains supplementary material available at 10.1007/s00484-024-02622-8.

## Introduction

Thermal stress is a global challenge. Rising temperatures and shifting climatologies driven by climate change are increasing heat stress and decreasing cold stress conditions, and could render some regions too hot for human habitation by 2100 (Di Napoli et al. 2023; Antonescu et al. 2021; Coffel et al. 2018; Lorenz et al. 2019). While research asserts that reductions in cold stress are unlikely to meaningfully impact mortality, dramatic increases in the number of mortalities from heat stress are expected (Ebi and Mills 2013; Gasparrini et al. 2017; Staddon et al. 2014; Kinney et al. 2015; Matthews et al. 2017). Across Australia heat wave days are expected to at least double by 2100, and increase by at least sixfold along the populous east coast (Nishant et al. 2022). To best prepare for and adapt to these challenges, projections of thermal stress that account for regional climate change and the impacts of local geography are needed.

Human thermal stress is driven by four meteorological factors: air temperature, relative humidity, wind speed, and mean radiant temperature (T_mrt_) (Höppe 1999). Historically, measurements of thermal stress have generally avoided accounting for T_mrt_ due to challenges of measurement and modelling Kántor and Unger (2011). T_mrt_ measures the sum of short ($\lambda$=0.3–3 $\mu$m) and long wave ($\lambda$=3–100 $\mu$m) radiation load on a person at a specific location and time, and assigns a temperature in Kelvin or Celsius to this load. This is the temperature of a black body sphere enclosing a person that would provide the same radiation load as measured in the environment (Kántor and Unger 2011). T_mrt_ may range from above ${80}^{\circ }$C to below ${-20}^{\circ }$C. Shading effects in strong sunshine can reduce T_mrt_ values by ${30}^{\circ }$C or more (Crank et al. 2020; Acero et al. 2021; Middel and Krayenhoff 2019; Thorsson et al. 2014). Advances in modelling have seen the emergence and adoption of thermal stress metrics encompassing all four meteorological variables, such as the Universal Thermal Climate Index (UTCI) and Physiologically Equivalent Temperature (PET), which have shown a potential to improve the identification of dangerous thermal conditions compared to simpler metrics (Błażejczyk et al. 2013; Höppe 1999; Di Napoli et al. 2019). In this paper, we use the UTCI, which considers a set of meteorological conditions, models how an idealised human walking at 4km h^-1^ would respond, and determines an equivalent air temperature under still, cloudy conditions (Błażejczyk et al. 2013).

Thermal stress has been projected using a variety of data sources, at multiple spatial and temporal scales (Petersson et al. 2019). Many projections have been performed using daily statistics or restricted variables, without accounting for the impacts of radiation. For example, Casanueva et al. (2019), Coffel et al. (2018); Schwingshackl et al. (2021) all projected Wet Bulb Globe Temperature (WBGT) using daily temperature maxima, and daily mean humidity. These are robust methods at daily frequencies, but heat stress can have serious health impacts in less than an hour, and the four meteorological variables that contribute to heat stress can vary significantly on hourly timescales (Bernard and Ashley 2009). Such impacts are observed during sport, where exertion and clothing can play a role Smith et al. (2018); Costa et al. (2020); Armstrong et al. (2010). Hourly calculations of thermal stress can therefore provide assessments of heat impacts more relevant to human physiology. Vargas Zeppetello et al. (2022) highlighted this, projecting Heat Index (HI) values using daily maximum temperature and monthly mean specific humidity, commenting that future work should consider more detailed variations in humidity, given its impact on heat stress. Hourly calculations also allow for more nuanced decision-making around activity safety and precautions at different times of day.

Thermal stress projections informed by radiation at hourly time scales have been performed, but often at very coarse spatial scale (e.g., kilometre grid cell size) (Paranunzio et al. 2021; Brecht et al. 2020; Katavoutas et al. 2022; Bal and Kirchner 2023). While these methods are robust at their scale, they cannot account for variations in thermal stress driven by fine-scale objects, while conditions can change from safe to dangerous at metre scale (Weeding et al. 2023). The first projections to address the limitations imposed by the relatively coarse temporal and spatial scale in previous models were published by Thorsson et al. (2011). The projections were calculated at hour and metre scale, using a historical analogue technique due to the requirement for “realistic, multi-variable climate inputs at sub-daily time resolution” (Thorsson et al. 2011, pg. 327) at a time when the first multivariate bias correction techniques were still under development. However, the use of an analogue technique rather than bias correction meant their projections could not contain conditions more extreme than historically recorded. Citing this issue, Rayner et al. (2015) developed a change factor technique using comparisons of ranked daily statistics to derive hourly change factors. This technique is in current use, but as Rayner et al. (2015) notes, it assumes that proportional changes to solar radiation under climate change will be consistent throughout the day and that change factors can be independently applied to temperature and radiation without consideration of relationships between the variables (Thorsson et al. 2017; Wallenberg et al. 2023). Such relationships can have a significant influence when calculating an index such as the UTCI, based on four variables, each with cyclical and stochastic influences. Bias correcting the individual variables of a compound metric without accounting for relationships between the variables can increase biases and should be avoided Zscheischler et al. (2019).

**Fig. 1:**
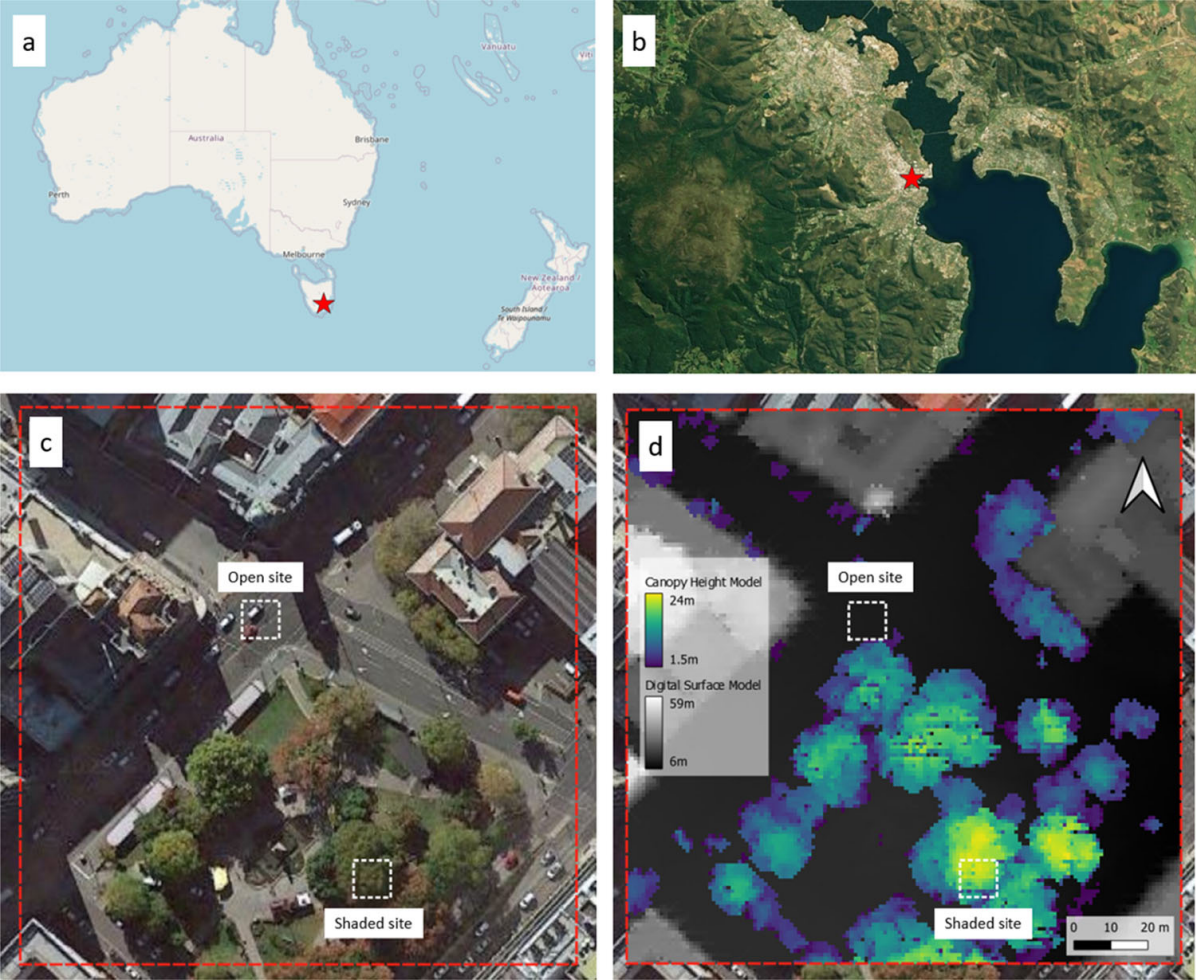
**a** Hobart’s location in regional context, **b** the study site in local context, **c** 150 m $\times$ 150 m study site outlined in red, with open and shaded sub-sites highlighted - Hobart, Tasmania (${42.883}^{\circ }$S  ${147.330}^{\circ }$E), **d** the site shown with a transparent overlay of the digital surface and canopy models (Google 2023; OpenStreetMap Contributors 2023; Microsoft 2023)

This study aims to provide a detailed understanding of changing thermal stress patterns incorporating modern measurements, bias correction techniques, and hourly projections to assess the impact of climate change on thermal stress at human scales. To achieve these aims, we present a case study of central Hobart, Australia, modelled at metre scale on an hourly basis for the years 2040–2059 under Representative Concentration Pathway (RCP) 8.5. Projections of meteorology were taken from six dynamically downscaled models at 5 km scale. Data at 1.5 km scale from the Bureau of Meteorology Atmospheric high-resolution Regional Reanalysis for Australia-Tasmania (BARRA-TA) from 1990 to 2005 was used to apply a multivariate bias correction to the projections (Su et al. 2021). To calculate UTCI values, T_mrt_ values were first generated from the described projections at metre scale using the SOlar and LongWave Environmental Irradiance Geometry model (SOLWEIG) (Lindberg et al. 2008). UTCI values were then calculated using the thermofeel Python package (Brimicombe et al. 2022). The impacts of bias correction were analysed by hour of day and week of year and for conservation of seasonal temperature trends. Projected changes to air temperature and UTCI were analysed by hour of day and month of year simultaneously, before changes to the 5^th^, 50^th^, and 95^th^ percentiles of maximum and minimum UTCI by hour were explored. Mean hours of any heat or cold stress were inspected by month of the year and hour of the day, followed by an analysis of cumulative strong and very strong heat stress hours for annual periods beginning with winter.

## Data and methods

### Site details and climate

This study took place in Hobart, Australia (Fig. 1a). Hobart has a temperate oceanic climate (Cfb), with a mean rainfall of 565.3 mm, a mean maximum temperature of ${17.6}^{\circ }$C and a mean minimum of ${9.0}^{\circ }$C. The warmest weather occurs in January with a mean maximum of ${22.7}^{\circ }$C, while the coldest occurs in July with a mean minimum of ${5.2}^{\circ }$C (Beck et al. 2018; Bureau of Meteorology 2023). These comparatively dry and mild conditions for Tasmania are due to the city’s eastern estuarine location and the westerly shelter provided by kunanyi/Mt Wellington (1271 m)(Fig. 1b). Northwesterly winds are predominant year-round, interrupted by cool southeasterly afternoon sea breezes in warmer months. Warm summer conditions are subject to rapid changes, due to passing cold fronts between high-pressure systems. Hobart has experienced relatively slow climate change for Australia—from 1940 to 2007, Hobart’s annual mean temperature increased by ${0.1}^{\circ }$C per decade, and the annual maximum and minimum temperatures increased by ${0.08}^{\circ }$C and ${0.11}^{\circ }$C per decade respectively (Corney et al. 2010; Campbell et al. 2019; Bureau of Meteorology 2023; Weeding et al. 2023).

In this study, we used the same central Hobart site as in Weeding et al. (2023). The site comprises a 150 m $\times$ 150 m square centred on the Elizabeth and Macquarie street intersection (Fig. 1). Hobart has global, national, and local relevance, as Tasmania has been identified as a favourable migration centre under catastrophic climate change, and is already seeing climate-driven migration from mainland Australia (King and Jones 2021; Osbaldiston et al. 2020; Osbaldiston 2022). The site contains hard-surfaced open areas, a vegetated park, and an urban canyon-oriented NW-SE. We generated a height raster of the site from publicly available lidar data in point cloud form (Land Tasmania 2015, 2018). Using the method described in the Urban Multi-scale Environmental Predictor (UMEP) manual (Lindberg et al. 2020, 2018), the following raster layers were produced at 1 m scale: (i) a digital elevation model (DEM) containing ground elevations, (ii) a digital surface model (DSM) containing building and ground elevations, and (iii) a canopy height model (CHM) containing vegetation elevations.

### Data sources

Historical simulations and projections of meteorological data were taken from six General Circulation Models (GCMs), modelled under Representative Concentration Pathway (RCP) 8.5 and dynamically downscaled to 5 km by the University of Tasmania and the Victorian Climate Projections 2019 project (Harris et al. 2020; Clarke et al. 2019) (Table S1). Six models were chosen to represent the range of warming seen for Australia in the Coupled Model Intercomparison Project (CMIP) 5. Dynamical downscaling was performed using the CSIRO’s Conformal Cubic Atmospheric Model (CCAM) (McGregor and Dix 2008). It should be noted that CCAM was run without ocean feedback mechanisms being accounted for, but that the Urban Climate and Energy Model (UCLEM) was included, which ensures macro-urban climate phenomena are captured in the modelling (Lipson et al. 2018). We selected historical outputs from 1990 through 2005 to overlap with BARRA-TA, and projected outputs for 2040 through 2059. Importantly, the BARRA-TA reanalysis contains nearby urban station data. The four meteorological variables required to calculate the UTCI were extracted from the models from the grid cell containing our study site (Table S2).

### Bias correcting

Climate models display biases when compared to observations, and therefore undergo bias correction to improve projections. Model values are altered to align statistical properties of model outputs with corresponding observations (François et al. 2020). Bias corrections have generally been performed on individual variables in isolation, but attempts to project multivariate indices such as the UTCI have shown that combining multiple variables that have been bias-corrected in isolation can lead to physically inconsistent results. This has led to the development of multivariate bias correction techniques, which adjust model values to align statistical properties of individual variables with observations while accounting for the relationships present between observed variables.

In this study, we applied a diurnal version of Cannon’s N-dimensional multivariate bias correction (MBCn) process 2018, guided by the work of Faghih et al. (2022). The aim of MBCn is to correct the variables while replicating the relationships between them. This first entailed the individual bias correction of each of the four variables (Table S2) using Quantile Delta Mapping (QDM) (Cannon et al. 2015). Variables were grouped by both hour of the day and week of the year. Weeks 52 and 53 were combined to increase the number of data points in the associated groups. Adjustments were multiplicative for all variables. Strong annual cycles present in downward shortwave radiation data in combination with varied amounts of cloud between the reanalysis and model outputs occasionally resulted in the calculation of invalid adjustment factors, driven by the presence of zeros in a set of model outputs and not in the reanalysis, or vice versa. Therefore, any infinite adjustment factors for downward shortwave radiation were set to zero. This occurred for 0.065% of adjustment factors. After individual correction, the MBCn algorithm was applied on data grouped by hour of the day and day of the year. This grouping was used to retain the diurnal and annual cycles as strongly as possible. The MBCn algorithm aims to replicate the multidimensional distribution from the observed data by reordering values from each of the four individually corrected variables within their time groups. This reordering process is determined using an iterative process using matrix algebra and QDM. Technical details can be found in Cannon (2018), and our implementation in Weeding and Love (2023).

### Modelling T_mrt_ and UTCI

T_mrt_ and UTCI were modelled at 1.5 m above ground at the central points of a 1 m scale grid, spanning the 150 m $\times$ 150 m site. To balance the speed of computation with model accuracy, the site was divided into nine 50 m $\times$ 50 m tiles. A 50 m buffer for each tile was included when modelling, ensuring that the influence of features around the edge of the site was not ignored. This allowed modelling to be performed on the buffered tiles in parallel before results from each of the nine tiles were concatenated to cover the 150 m $\times$ 150m site. Land cover types for each grid point were determined by identifying building footprints and automatically classifying remaining surfaces (paved, bare soil, grass, and buildings) using intensity data from lidar.

**Fig. 2:**
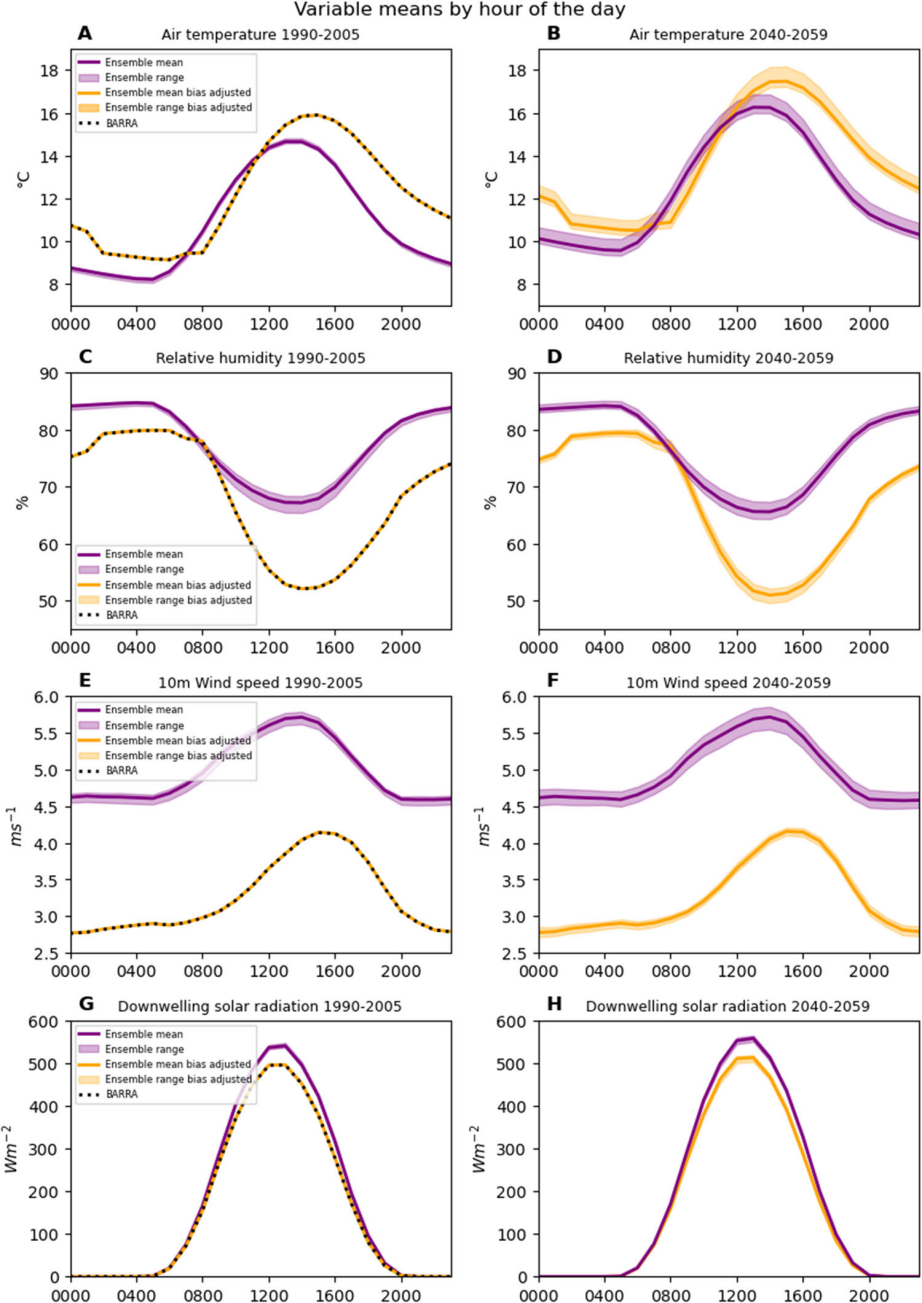
Means by hour of the day of air temperature, relative humidity, 10 m wind speed, and downwelling solar radiation for BARRA-TA, raw model outputs, and bias-corrected model outputs. The range of means across the six model ensemble is shown by the pale colouring, while the mean of the ensemble is shown by the solid lines

For each hour, mean T_mrt_ was modelled using SOLWEIG v2019a as part of the Urban Multi-scale Environmental Predictor (UMEP v1.3) package (Lindberg et al. 2018). A detailed exposition of SOLWEIG’s processes can be found in Lindberg et al. (2016). SOLWEIG calculates T_mrt_ at each grid centre by determining the shortwave and longwave radiation incident on a cylindrical model of a person at centre of the tile. These radiation fluxes are determined by the interaction of direct and diffuse solar radiation with built surfaces and vegetation, as well as emissions from the sky and surfaces due to their temperature. The sum of these fluxes is the mean radiant flux density (S_str_), and can be used to calculate T_mrt_ as follows:

1 $$\begin{aligned} T_{mrt} = \root 4 \of {\frac{S_{str}}{\epsilon _{p}\sigma }}-273.15 \end{aligned}$$

where $\epsilon _{p}$ is the human body’s emissivity of 0.97 and $\sigma$ is Stefan-Boltzmann constant. Once T_mrt_ values have been calculated for each individual tile, hourly UTCI values can then be calculated. For each timestamp and grid, individual T_mrt_ values and the site-wide meteorology data are used to calculate the thermal stress that would be experienced under those conditions by a person walking at 4 km h^-1^ producing 135 W of metabolic heat. To give a UTCI value, this stress level is then equated to an air temperature under reference meteorological conditions: T_mrt_ equal to $T_{air}$, relative humidity of 50%, and a wind speed of 0.5 m/sec at a height of 10 m (Błażejczyk et al. 2013). UTCI values and stress categories are given in Table S3. In this work, we calculated UTCI with the widely used operational procedure via the thermofeel Python package, which introduces a root mean square error (RMSE) of ${1.1}^{\circ }$C compared to the computationally intensive but definitive calculation using the UTCI-Fiala multi-node model (Bröde et al. 2012; Brimicombe et al. 2022). This is less than the expected error from calculating UTCI from modelled radiation and is not considered large enough to be of concern when modelling outdoor thermal stress (Gál and Kántor 2020; Weihs et al. 2012).

**Table 1:** Ensemble variable means and stress conditions over the site between 1990–2005 and 2040–2059 after bias correction

Variable (units)	Mean (1990–2005)	Mean (2040–2059)	Net change
Air temperature (^∘^C)	12.1	13.5	1.4
Relative humidity (%)	68.1	67.4	$-$0.7
Wind speed ($ms^{-1}$)	3.3	3.3	0.0
Downwelling solar radiation ($Wm^{-2}$)	154.5	159.2	4.7
UTCI (^∘^C)	7.6	9.4	1.8
Stress conditions	% of hours	% of hours	Change factor
	(1990–2005)	(2040–2059)	
Any heat stress at site	6.09	8.76	1.44
($$UTCI_{\max } > 26$$)			
Site-wide heat stress	0.46	1.00	2.18
($$UTCI_{\min } > 26$$)			
Any no stress at site	89.58	92.22	1.03
Site-wide no stress	70.93	74.35	1.05
Any cold stress at site	22.98	16.89	0.74
($$UTCI_{\min } < 0$$)			
Site-wide cold stress	9.96	6.78	0.68
($$UTCI_{\max } < 0$$)			

**Fig. 3:**
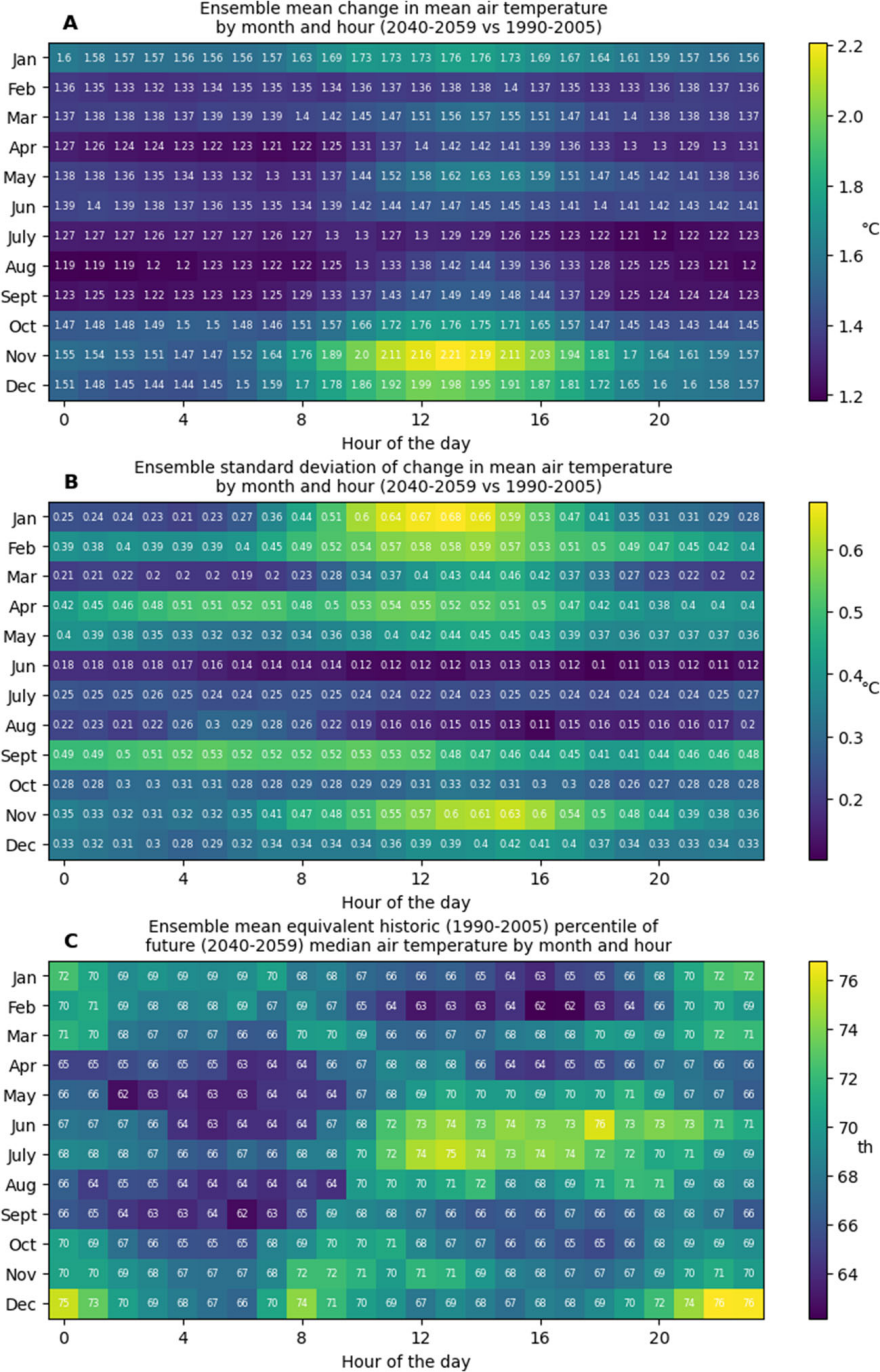
**A** Bias-corrected ensemble means of change in mean air temperature from 1990–2005 to 2040–2059 by hour of day and day of year. **B** Bias-corrected ensemble standard deviations of change in mean air temperature from 1990–2005 to 2040–2059 by hour of day and day of year. **C** Bias-corrected ensemble mean 2040–2059 median air temperatures expressed as 1990–2005 percentiles

**Fig. 4:**
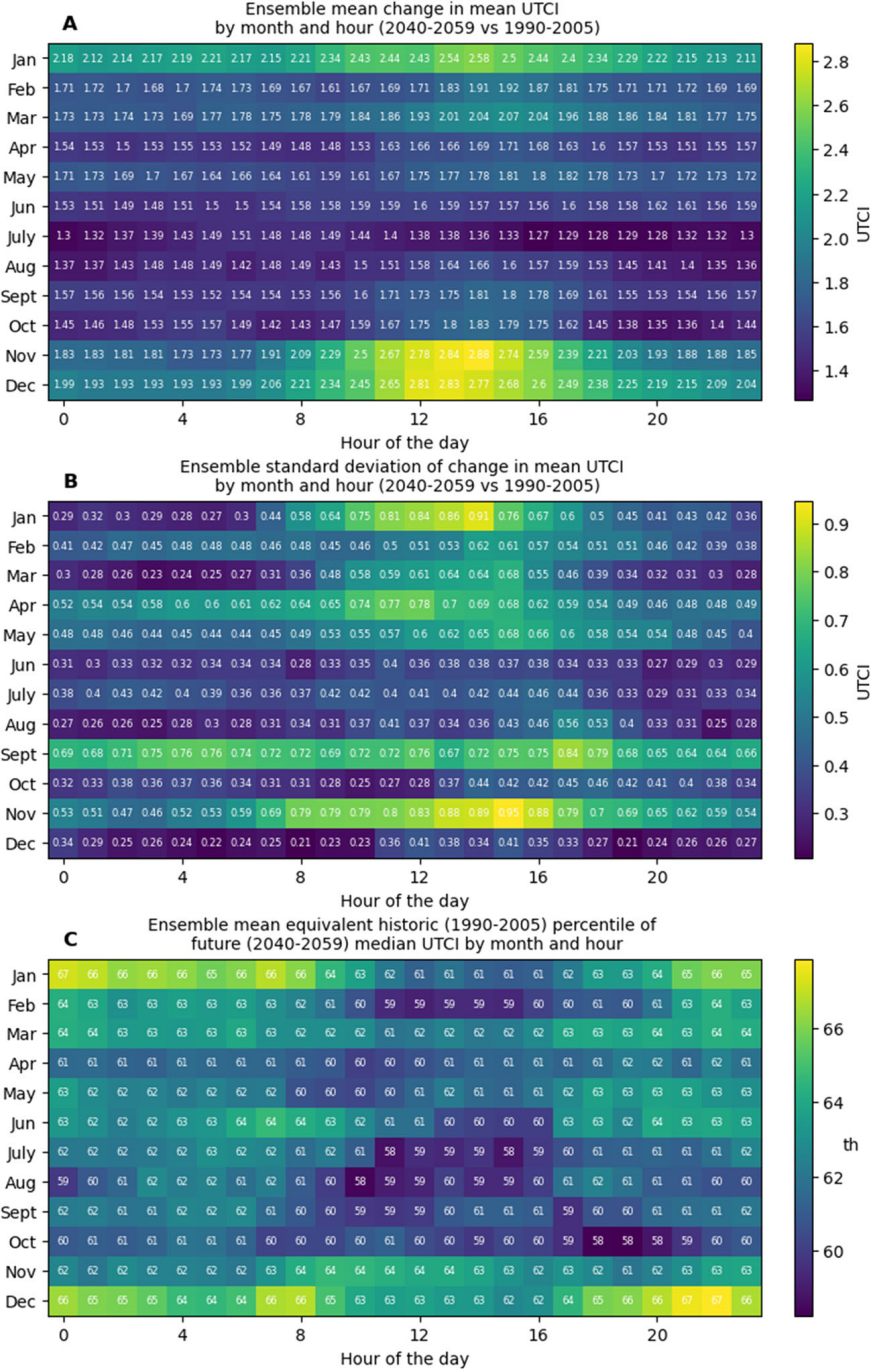
**A** Bias-corrected ensemble means of change in mean UTCI from 1990–2005 to 2040–2059 by hour of day and day of year. **B** Bias-corrected ensemble standard deviations of change in mean UTCI from 1990–2005 to 2040–2059 by hour of day and day of year. **C** Bias-corrected ensemble mean 2040–2059 median UTCIs expressed as 1990–2005 percentiles

## Results

The ensemble of models and BARRA-TA contained different hourly means and diurnal cycles over the historical period (1990–2005) (Fig. 2). Diurnal cycles of air temperature and wind speed peaked at later hours in BARRA-TA compared to the ensemble. Wind speeds in BARRA-TA were lower than in the ensemble. The diurnal cycles of solar radiation were synchronous between BARRA-TA and the ensemble and of similar magnitudes. Historical means and diurnal cycles were accurately altered by bias correction. For the future period (2040–2059), bias correction resulted in diurnal and annual cycles similar in shape to those seen in the BARRA-TA and the historical bias-corrected data (Figure S2). Seasonal mean air temperature change signals were preserved by the bias correction process (Figure S1). For each season, trends across the models agreed to within $\pm$ ${0.5}^{\circ }$C but for HadGEM2-CC, which showed greater warming than other models except in winter. Seasonal temperature trends were slower than in the same modelling for Victoria, where ensemble median warming between the historic and future periods was more than ${2}^{\circ }$C for all seasons except winter (Clarke et al. 2019).

Changes in mean values between the historic and future periods were largest for air temperature and UTCI, while no change in mean wind speed was observed. These changes manifested in reductions in cold stress conditions, and increases in no stress and heat stress conditions (Table 1). Increases in air temperature and UTCI occurred throughout the seasons (Figure S3). Changes in mean air temperatures by month and hour displayed annual and diurnal patterns (Fig. 3A). Warming was generally greater during the day and during warmer months. Early afternoon in November showed the greatest warming. Agreement between the models varied month on month, and lower levels of agreement coincided with both high and low levels of warming (Fig. 3B). Future median air temperatures expressed as historic percentiles generally showed stronger warming for the daytime than the night-time during the cooler months (Fig. 3C). Diurnal patterns were less evident in the warmer months. Changes in mean UTCI by month and hour, and model agreement on these changes showed similar patterns to air temperature (Fig. 4A and B). Large increases for November and December daytime hours were more prominent in UTCI than in air temperature, due to coincident increases in downwelling solar radiation. Future median UTCI values expressed as historic percentiles were generally lower than for air temperature and displayed different temporal patterns (Fig. 4C). Increases were generally larger during night-time, with the largest changes occurring during December and January.

Future 5^th^, 50^th^, and 95^th^ percentile site-wide maximum and minimum UTCIs were higher than their historical equivalents for all hours of the day (Fig. 4). While historic 95^th^ percentile site-wide maximums were never classified as greater than moderate heat stress, future 95^th^ percentile site-wide maximums from 1100 to 1600 h were of strong heat stress conditions. Increases to 50^th^ percentile site-wide maximums displayed a diurnal cycle. All historic 5^th^ percentile side-wide minimum UTCIs were of moderate cold stress conditions, while future 5^th^ percentile site-wide minimum UTCI values for 1300 to 1400 h were of no stress conditions. Increases to 50^th^ percentile site-wide minimums also displayed a diurnal cycle. The proportions of extreme heat stress hours increased by 450% and 268% for maximum and minimum UTCIs respectively, between historic and projected outputs.

Annual mean hours of any level of heat stress across the entire site were projected to increase for September through May, with no periods of site-wide heat stress occurring during winter in both the historic and future periods (Fig. 6A). Annual mean hours of any level of site-wide cold stress were projected to decrease for all months in the future period but never reached zero for any month (Fig. 6B). Annual mean hours of any level of site-wide heat stress by hour were projected to increase for all hours except 0200 through 0600 (Fig. 6C). No instances of any level of site-wide heat stress were reported for either the historic or future periods for the hours 0200 through 0600, while future projections reported instances of heat stress for the hours 0000, 0100, and 0700. Annual mean hours of any level of site-wide cold stress were projected to decrease for all hours of the day (Fig. 6D). Over the historic period, all combinations of hour and year reported some instances of any level of site-wide cold stress. However, for future combinations of hour and year, there were eight instances where no site-wide cold stress was projected, all during the hours of 1200 through 1400, across five different years and four models.

Total hours from June through May of any strong heat stress at the site were projected to increase substantially, with an increase in the median number of hours of more than 70% and the lowest projected total number of hours for the future period of 95 h. Only two future annual totals were less than the median historic total (Fig. 7A–D. The median day of the year at which any strong heat stress first occurs was projected to change from the 30^th^ of October to the 10^th^ of October. Cumulative hours from June through May of any very strong heat stress at the site were projected to increase in greater proportion than strong heat stress, with the median number of hours increasing 125% and all future years containing some very strong heat stress. The median day of the year at which any very strong heat stress first occurs was projected to change from the 3^th^ of December to the 21^th^ of November, ignoring the two historic model run years that contained no very strong heat stress.

## Discussion

### Consequences of bias correction

Errors in model outputs compared to observations have made bias corrections almost obligatory for climate change impact studies (Faghih et al. 2022; Zscheischler et al. 2019). However, the assumptions that corrections make cannot be ignored. Bias correcting at sub-daily timescales is a developing field and can have major impacts on compound metrics such as the UTCI, which combines four diurnal cycles. Figure 2 demonstrates how rigidly the bias correction forces the diurnal cycles present in BARRA-TA onto the model outputs. This is not necessarily incorrect, but does assume that BARRA-TA’s diurnal cycles will be valid for 2040–2059, or are at least better than those in the ensemble. Similarly strong correction and assumption is shown in Figure S2, in particular for wind speed and radiation. BARRA-TA contains a weaker annual cycle for wind speed than the ensemble, and this manifests in the bias-corrected projections. This weaker annual cycle is still present when the entire BARRA-TA time series (1990–2019) is analysed, instead of the overlapping 1990–2005 data. Compared to the model ensemble, BARRA-TA also contains lower mean downwelling solar radiation in February and December, which is reflected in the bias-corrected projections. When the entire BARRA-TA time series (1990–2019) is used, the difference in mean downwelling solar radiation between BARRA-TA and the model ensemble is reduced, demonstrating that the use of a longer historic period for bias correction would have tangible impacts on the corrected projections.

When interpreting trends in mean air temperature from the historic to future periods by month and hour (Fig. 3), it is important to remember that these trends are ensemble means and that the MBCn method is inherently trend preserving. This means the observed trends originate in the models, and were not imposed by bias correction. The strong early afternoon warming trends in November air temperatures provide an example (Fig. 3A). An analysis of November trends in the individual models before bias correction revealed the major driver of these warming trends to be a very strong diurnal cycle in the trends of the HadGEM2-CC model. For HadGEM2-CC, night-time trends averaged ${2.2}^{\circ }$C, while midday trends from 1100 through 1600 h averaged ${3.2}^{\circ }$C. MIROC5 and ACCESS 1–0 showed similar diurnal cycles in their warming trends, but at lower magnitudes—trends for 1100 through 1600 h averaged ${2.4}^{\circ }$C and ${2.43}^{\circ }$C, respectively. The remaining three models showed little in the way of diurnal cycles in their trends and contained no warming trends greater than ${1.8}^{\circ }$C at any time of day. This disagreement within the ensemble is reflected in the standard deviation data for November (Fig. 3B). The same analysis showed that the largest standard deviations for air temperature trends were driven by opposing January diurnal cycles: HadGEM2-CC contained its highest warming signals during the daytime, while MIROC5 and NorESM1-M contained their lowest warming signals. Similar patterns were observed in UTCI trends (Fig. 4A and B). The most noticeable difference between air temperature and UTCI trends is the very strong mid-afternoon increasing trend in UTCI seen in both November and December. An analysis of the four contributing factors revealed this was driven by simultaneous trends in air temperature and downwelling solar radiation.

**Fig. 5:**
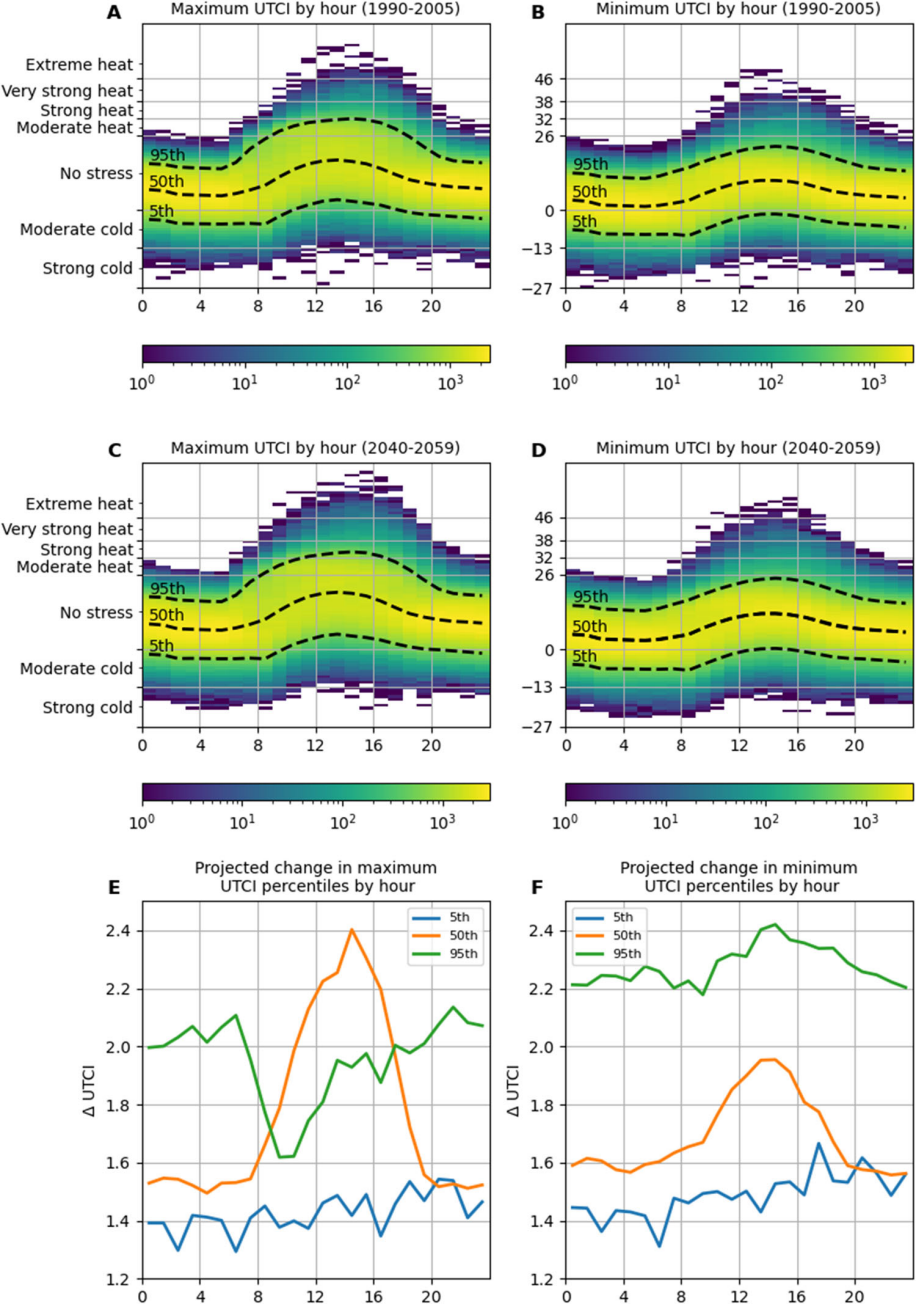
**A** All 1990–2005 bias-corrected ensemble site-wide UTCI maximum values by hour of the day, overlaid with 5^th^, 50^th^, and 95^th^ percentiles. **B** All 1990–2005 bias-corrected ensemble site-wide UTCI minimum values by hour of the day, overlaid with 5^th^, 50^th^, and 95^th^ percentiles. **C** All 2040–2059 bias-corrected ensemble site-wide UTCI maximum values by hour of the day, overlaid with 5^th^, 50^th^, and 95^th^ percentiles. **D** All 2040–2059 bias-corrected ensemble site-wide UTCI minimum values by hour of the day, overlaid with 5^th^, 50^th^, and 95^th^ percentiles. **E** Changes to 5^th^, 50^th^, and 95^th^ percentiles of bias-corrected ensemble site-wide UTCI maximums by hour of the day. **F** Changes to 5^th^, 50^th^, and 95^th^ percentiles of bias-corrected ensemble site-wide UTCI minimums by hour of the day

**Fig. 6:**
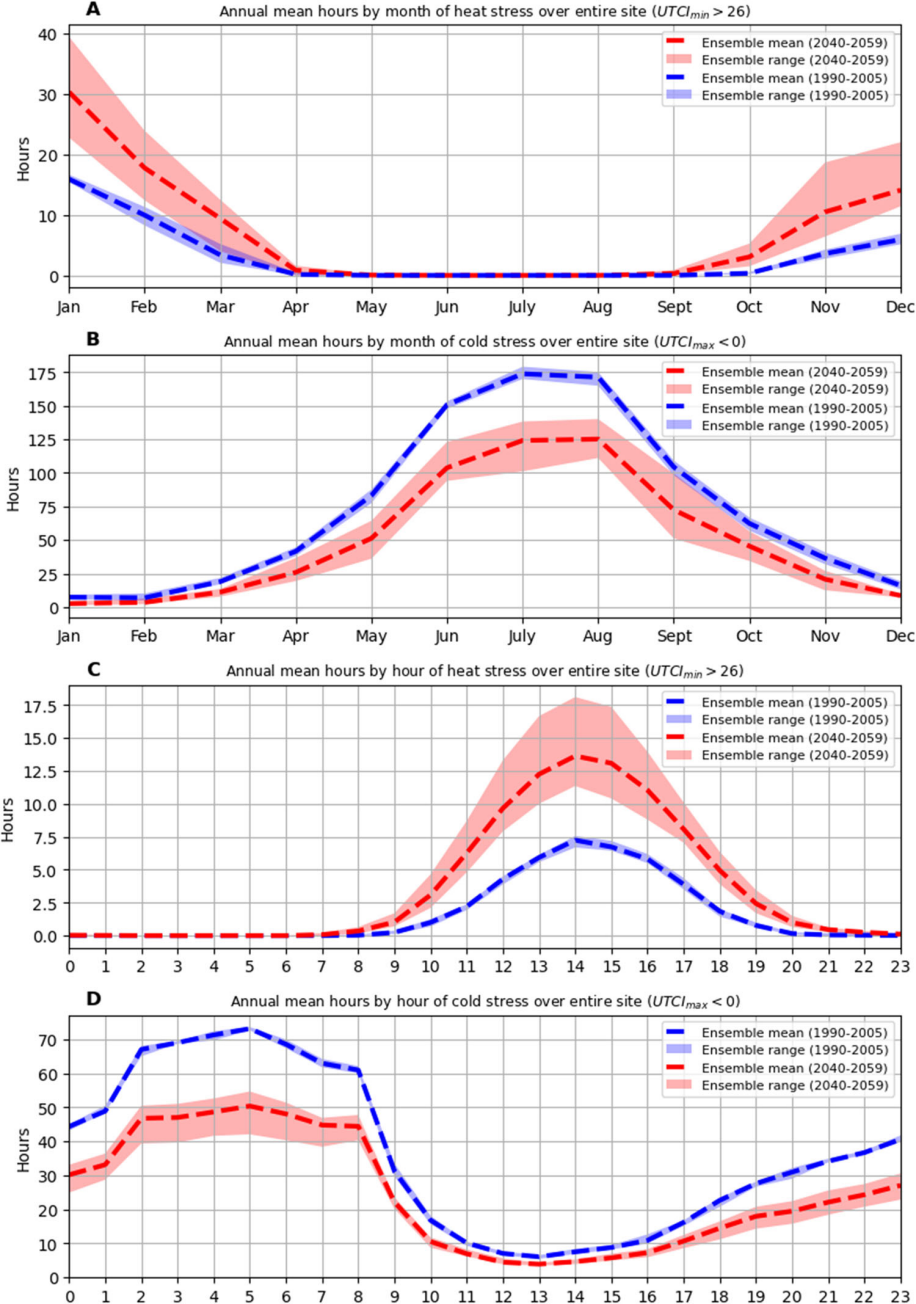
**A** Annual mean hours of site-wide heat stress conditions by month of the year for 1990–2005 and 2040–2059 bias-corrected ensembles. **B** Annual mean hours of site-wide cold stress conditions by month of the year for 1990–2005 and 2040–2059 bias-corrected ensembles. **C** Annual mean hours of site-wide heat stress conditions by hour of the day for 1990–2005 and 2040–2059 bias-corrected ensembles. **D** Annual mean hours of site-wide cold stress conditions by hour of the day for 1990–2005 and 2040–2059 bias-corrected ensembles

**Fig. 7:**
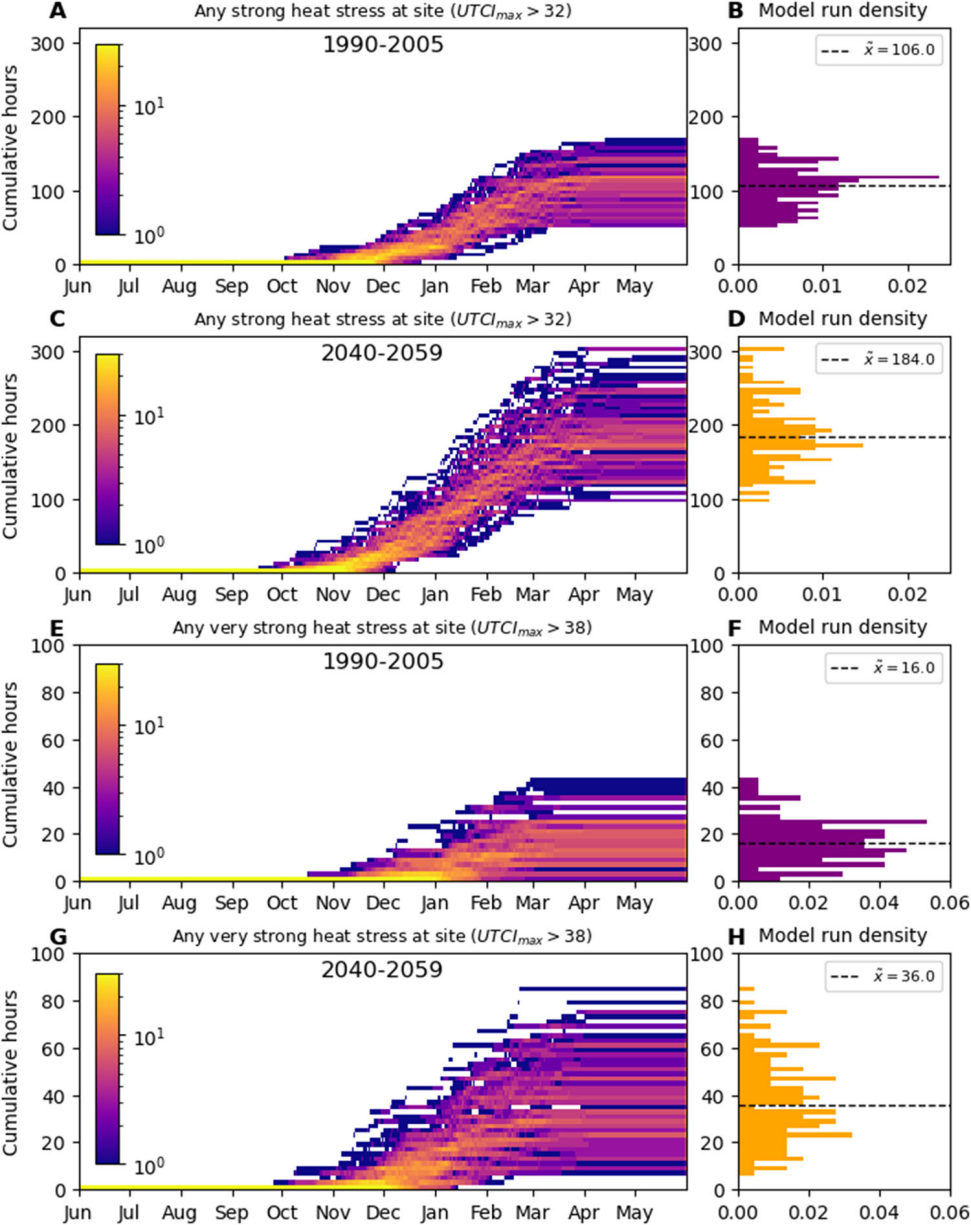
Plots **A**, **C**, **E**, and **G** show cumulative hours of any occurrence at the site of a category of heat stress for a June through May year, plotted by year and model. Plots **B**, **D**, **F**, and **H** show the corresponding density histogram of the totals

### Interpreting the heat stress data

When interpreting Figs. 5, 6, and 7, it is important to consider the relationship between shading and the plotted statistics. UTCI site maximums describe open conditions during the daytime and vegetation-shaded conditions at night, due to the presence of shortwave radiation and the insulating effect of the canopy respectively. Conversely, UTCI site minimums describe locations shaded by vegetation and buildings during the day and open areas at night. Therefore, Fig. 5A–D illustrates the impact of shade in reducing stress extremes throughout the day, as described in Weeding et al. (2023). This dampening effect is also visible in the changes to the 50^th^ percentiles in Fig. 5E and F, where the change signal for site minimum UTCI is muted during daylight hours compared to the site UTCI maximums. Interestingly, the 95^th^ percentiles show greater increases for the site UTCI minimums than maximums. These minimums will occur under shade during hours of high air temperature. This illustrates that these increases in air temperatures have slightly greater net effects on stress conditions under the shade than in the open.

Projected changes for Hobart are comparable to those in the literature. An increase in mean UTCI of ${1.8}^{\circ }$C over 35 years equates to ${0.05}^{\circ }$C per year (Table 1). This is equal to recent observations for Cfb climates in Europe and comparable to RCP8.5 projections of Cfb climates in Germany (Antonescu et al. 2021; Brecht et al. 2020). The projection of an overall increase in the number of no stress conditions in the near future has also been projected in four contrasting European cities, albeit at a broader spatial scale ($0.11^{\circ }$ grid) (Katavoutas et al. 2022). In both our work and that of Katavoutas et al. (2022) this is due to reductions in the occurrence of cold stress conditions dominating increases of heat stress conditions, with changes of comparable magnitude. However, thermally neutral conditions were more common in Hobart than in any of the four European cities.

Figures 5, 6, and 7 describe a markedly different thermal environment in Hobart for 2040–2059 than that experienced during 1990–2005. Higher UTCI values, recorded earlier and later in the day, and greater average hours of heat stress over the majority of hours of the day and months of the year will likely change perceptions of seasons and hours of the day, place acclimatisation demands on citizens, and drive behaviour changes. For example, from the perspective of hours of heat stress across the entire site—that is, heat stress occurring even in the shade—future Marches and Novembers are equivalent to historic Februaries (Fig. 6). Similarly, when considering hours of the day, citizens can expect more future hours of heat stress at 1700 than historically experienced at 1400, the most stressful hour of the historic period. In terms of cold stress hours, future Junes are equivalent to historic Septembers, and future Julys and Augusts are halfway between historic Junes and Septembers. From an hourly perspective, the largest reductions in mean cold stress hours were observed during the night, with future early morning stress levels closer to historical levels around midnight than early morning.

Considering cumulative stresses, Fig. 7 shows citizens can expect to experience approximately double the number of hours some strong or very strong heat stress in open locations from one winter to the next for 2040–2059 compared to 1990–2005, and expect heat stress conditions to begin almost three weeks earlier. Perhaps the most striking illustration of the changes projected for 2040–2059 is that the median number of hours of strong heat stress (184) is 18 h greater than the maximum number of strong heat stress hours reported for 1990–2005. Almost as striking is that for very strong heat stress, only four out of 60 historic model run years contain more hours than the projected future median. These statistics, and Fig. 7 are of particular relevance for those working outdoors in urban environments, as they indicate the conditions they will be exposed to, and consequently have to plan for. It is important to note that the UTCI is calibrated for someone performing light activity in regular clothing, and heat stress levels will be amplified for those performing physically strenuous work or wearing highly insulating clothing. This is a significant point that should be emphasised when raising awareness of the dangers of heat stress.

Figures 3C and 4C present novel views of future temperature and thermal stress levels in recent context. These plots reveal patterns that are not evident when viewing mean changes, and emphasise how projected median conditions compare to those people have previously acclimatised to. For temperature, the largest relative changes occurred in summer and winter, but at opposing times of day. Summer temperatures show higher relative changes from the late evening through to mid-morning, with the greatest changes around 0000 and 0800 h, while for winter, daytime temperatures showed the largest changes. However, when considering relative changes in UTCI, we see an overall pattern of greater changes during warmer months and at night-time and a lower and narrower range of changes. When interpreting these changes and how they will change living conditions in the future Hobart, it is important to remember they are based on median conditions, and do not describe changes to extremes. This is evident in Fig. 5, which shows changes for the 5^th^, 50^th^, and 95^th^ percentiles are markedly different.

The changes above describe dramatic shifts in terms of planning outdoor work and recreation and have the potential to significantly alter behaviours and demands, especially in the construction, health and energy sectors. The UTCI is calculated for someone walking at 4 km h^-1^, and corresponding stress categories for a given UTCI value will therefore increase with greater levels of exertion as the body must deal with additional internally generated thermal energy. Humans can certainly adapt to higher thermal stress levels than those they are accustomed to as long as physiological thresholds are not breached, and there are sound arguments for developing adaptive stress categories based on UTCI baseline data at different locations (Lam and Lau 2018; Pantavou et al. 2018; Krüger et al. 2021). However, the impacts of different rates of acclimatisation are not well known and will vary significantly across society, depending on a wide range of health, economic, and cultural factors. Epidemiological monitoring of thermal conditions and their association with morbidity and mortality could inform healthcare practice and improve the prediction of demand. Ideally, comparisons of central Hobart’s future conditions could be made to other locations in Australia and beyond to inform adaptation.

### Sources of uncertainty and error

Outputs bias corrected using reanalysis data as the observations are inherently subject to the biases contained within the reanalysis. BARRA-TA’s known errors are described in Su et al. (2021), with perhaps the most relevant being reduced temperature extremes, and under-dispersed wind speeds for the Tasmanian region. The impact of reduced temperature extremes on the UTCI is relatively straightforward, reducing extreme UTCI values. The impacts of under-dispersed wind speeds are more complex but can be broadly described. Using under-dispersed data will result in speed reductions of wind speeds above average and speed increases for wind speeds below average. Therefore, for heat stress situations with above-average winds, bias correcting with BARRA-TA will likely overestimate heat stress, while for heat stress with below-average winds heat stress is likely to be underestimated. For cold stress situations, the opposite is true, with cold stress in above-average winds likely under-reported, and over-reported for below-average winds.

Errors will also arise from using 1.5 km scale BARRA-TA data at 1.5 m scale, as meteorology can be influenced by the urban environment at fine spatial scales. Vegetation can reduce air temperature and increase humidity through evapotranspiration and shading, all at scales not captured by BARRA-TA. These effects vary significantly with time, surface, and cover types, and while accurate localised modelling has been developed, its integration into thermal stress calculation workflows has not yet been achieved and is beyond the scope of this work (Rocha et al. 2022; Middel et al. 2021; Vulova et al. 2023). Therefore, it remains a contributing driver of errors. Trees, building surfaces and geometries will influence wind speeds, generally reducing them except in certain situations where urban canyons and building edges can cause localised speed increases (Grimmond and Oke 1999; Johansson et al. 2016; Yuan et al. 2017). A UMEP integrated wind model capable of modelling building and tree geometry as well as surface gradients is under development, and given reasonable computation times and validated results, should be adopted in future SOLWEIG based modelling and projections (Robinson et al. 2023; Bernard et al. 2023).

We generated elevation, surface, and canopy models from the latest available lidar data. While major change to the built environment of our site has not occurred since 1990 due to the predominantly heritage architecture at the site, changes to the vegetation community and morphology will have occurred due to planting, growth, and canopy management (Gulson 2007; Howard 2016). Vegetation changes will continue through to 2059—especially as the changing climate renders some species unviable—along with possible changes to the surrounding built environment which would alter shadow patterns and surface types. Therefore, computing our historic and projected outputs using a single set of elevation, surface, and canopy models will introduce errors. An example of such an error can be seen when comparing the site photograph and lidar data; a tree in the northernmost corner of the park is present in the lidar data, but not in the recent photograph (Fig. 1). Ideally, such errors could be reduced by using more frequently collected lidar survey data for the historic period, but this was not available. Making accurate predictions about changes to the site for the period 2040–2059 is likely insoluble. However, we do not consider this to reduce the validity of the outputs for the purposes of making realistic predictions of future climatic conditions. Errors may have been introduced when generating the canopy models. While some filtering was applied, additional filtering to remove the remaining linear features should be applied in future work (Lindberg and Grimmond 2011; Goodwin et al. 2009).

All trees were treated as evergreen, with the impacts of vegetation canopies on radiation modelled using SOLWEIG’s default values. These are shortwave and longwave transmissions through foliage of to 3% and 0%, respectively, vegetation albedo of 15%, and emissivity of 90% (Konarska et al. 2014; Oke 2002; Lindberg and Grimmond 2011). This will introduce errors, as the values will vary by solar position, species, canopy thickness and structure (Nyman et al. 2017; Hovi and Rautiainen 2020; Ribeiro da Luz and Crowley 2007). Errors of a similar nature arise from the simplified range of surface types modelled by SOLWEIG. Accurately resolving all these parameters via fieldwork is certainly impractical and likely impossible, and must acknowledge the associated errors, while noting that such errors are a consequence of working at spatial scales where individual trees and their canopies are resolved.

## Conclusions

This study presents projections of thermal stress at spatial and temporal scales relevant to human physiology, and an analysis of these stresses in the context of recent values for Hobart, Australia. These are the first hourly metre-scale projections of thermal stress driven by multivariate bias-corrected data, projecting thermal stress for 2040–2059 from a historical period of 1990–2005. Building on the work of Cannon (2018) and Faghih et al. (2022), we corrected four meteorological variables from six GCMs individually, as well as their interrelationships, using the BARRA-TA reanalysis (Su et al. 2021). We demonstrated that such a bias correction is able to powerfully correct means on multiple time scales, and accurately preserve mean seasonal trends. This bias-corrected data was used to drive modelling of thermal stress measured by the UTCI for a 150 m $\times$ 150 m site in central Hobart, Tasmania, Australia, with radiative processes resolved at 1 m scale by the SOLWEIG model (Lindberg et al. 2008). Plots of changes in mean air temperature and UTCI by hour of the day and month of the year revealed diurnal and annual patterns in both temporal trends and model agreement. We also analysed future median values by hour of the day and month of the year in terms of historical percentiles, revealing patterns not observed in the analysis of means. The ability of shade to mute extreme values and trends was evident when comparing site minimum and maximum UTCI values. Ways in which the future thermal environment might be interpreted and adapted to were considered, as well as the caveats that must be considered when using a powerful bias-correcting technique. Sources of error in the method were identified, in particular, those associated with working at such a fine spatial scale. The projections illustrated a significant change to Hobart’s thermal stress environment, with higher and more consistent numbers of hours of heat stress arriving earlier in the year and extending further throughout the day. These changes showed strong annual and diurnal patterns, demonstrating the value of modelling thermal stress at fine temporal scales.

### Supplementary Information

Below is the link to the electronic supplementary material.

Supplementary file 1 (tex 4 KB)

## Data Availability

This research was produced with publicly available data. The code and links to the data used to produce the bias correction and modelling are available in Weeding and Love (2023) at https://zenodo.org/records/8384767.

## Code availability

See above.
